# Bridging and Bonding Social Capital by Analyzing the Demographics, User Activities, and Social Network Dynamics of Sexual Assault Centers on Twitter: Mixed Methods Study

**DOI:** 10.2196/50552

**Published:** 2024-03-27

**Authors:** Jia Xue, Qiaoru Zhang, Yun Zhang, Hong Shi, Chengda Zheng, Jingchuan Fan, Linxiao Zhang, Chen Chen, Luye Li, Micheal L Shier

**Affiliations:** 1 Factor Inwentash Faculty of Social Work University of Toronto Toronto, ON Canada; 2 Faculty of Information University of Toronto Toronto, ON Canada; 3 Artificial Intelligence for Justice Lab University of Toronto Toronto, ON Canada; 4 Department of Sociology, Anthropology, Social Work, and Criminal Justice Seton Hall University South Orange, NJ United States

**Keywords:** social media, Twitter, sexual assault, nonprofits, Canada, violence, geolocation, communication

## Abstract

**Background:**

Social media platforms have gained popularity as communication tools for organizations to engage with clients and the public, disseminate information, and raise awareness about social issues. From a social capital perspective, relationship building is seen as an investment, involving a complex interplay of tangible and intangible resources. Social media–based social capital signifies the diverse social networks that organizations can foster through their engagement on social media platforms. Literature underscores the great significance of further investigation into the scope and nature of social media use, particularly within sectors dedicated to service delivery, such as sexual assault organizations.

**Objective:**

This study aims to fill a research gap by investigating the use of Twitter by sexual assault support agencies in Canada. It seeks to understand the demographics, user activities, and social network structure within these organizations on Twitter, focusing on building social capital. The research questions explore the demographic profile, geographic distribution, and Twitter activity of these organizations as well as the social network dynamics of bridging and bonding social capital.

**Methods:**

This study used purposive sampling to investigate sexual assault centers in Canada with active Twitter accounts, resulting in the identification of 124 centers. The Twitter handles were collected, yielding 113 unique handles, and their corresponding Twitter IDs were obtained and validated. A total of 294,350 tweets were collected from these centers, covering >93.54% of their Twitter activity. Preprocessing was conducted to prepare the data, and descriptive analysis was used to determine the center demographics and age. Furthermore, geolocation mapping was performed to visualize the center locations. Social network analysis was used to explore the intricate relationships within the network of sexual assault center Twitter accounts, using various metrics to assess the network structure and connectivity dynamics.

**Results:**

The results highlight the substantial presence of sexual assault organizations on Twitter, particularly in provinces such as Ontario, British Columbia, and Quebec, underscoring the importance of tailored engagement strategies considering regional disparities. The analysis of Twitter account creation years shows a peak in 2012, followed by a decline in new account creations in subsequent years. The monthly tweet activity shows November as the most active month, whereas July had the lowest activity. The study also reveals variations in Twitter activity, account creation patterns, and social network dynamics, identifying influential *social queens* and marginalized entities within the network.

**Conclusions:**

This study presents a comprehensive landscape of the demographics and activities of sexual assault centers in Canada on Twitter. This study suggests that future research should explore the long-term consequences of social media use and examine stakeholder perceptions, providing valuable insights to improve communication practices within the nonprofit human services sector and further the missions of these organizations.

## Introduction

### Use of Social Media by Nonprofit Organizations

Social media platforms, including Twitter (subsequently rebranded as X [X Corp]), have gained popularity among nonprofit advocacy organizations as essential tools for communication and public engagement [1,2]. Nonprofit organizations are increasingly recognizing the strategic value of social media in fostering public engagement, securing donations, disseminating information, recruiting volunteers, and raising awareness about social issues [3-8]. Today, most large and mid-sized nonprofit organizations actively maintain at least 1 social media account, underscoring the extensive use of social media within the nonprofit realm [9].

Twitter, for instance, offers nonprofit organizations a platform to create profiles, establish networks, and engage socially through features such as tweeting, sharing multimedia content, replying, and retweeting [10]. Recognized as a cost-effective means of consistently reaching a broader audience [11,12], Twitter proves especially valuable for nonprofit organizations, often facing limited financial resources and dedicated communication staff [12], including sexual assault centers, to actively engage with key stakeholders and spark meaningful conversations [13,14]. Moreover, engaging in dialogues with other Twitter users forms a central aspect of communication for these organizations, facilitating increased supporter involvement, knowledge dissemination, and the creation of supportive communities [15,16]. Nonprofit organizations have successfully captured their followers’ attention by regularly tweeting, responding to specific tweets, and retweeting other users’ content [2]. This social media engagement can be harnessed by nonprofit organizations to share educational information and advocate for social causes [17].

### Bridging and Bonding Social Capital

Social capital plays a critical role in understanding the effectiveness of nonprofit organizations, as it is embedded within their networks, enabling them to enhance their adaptive capabilities by consolidating shared interests and harnessing diverse resources [18,19]. Within the context of social capital, Putnam [20] distinguishes between 2 fundamental forms: bridging and bonding social capital. Bridging social capital encompasses the distant and weak connections between individuals from diverse backgrounds, facilitating information flow. This often manifests as nonmutual following relationships between organizations and a diverse public. In contrast, bonding social capital revolves around preexisting and robust ties that reinforce homogeneity among groups, fostering emotional and social support. An illustrative example of this is the mutual following relationships observed between similar organizations [21,22].

From a social capital perspective, relationship building is seen as an investment. It involves a complex interplay of tangible and intangible resources, both embedded within existing relationships and generated through the act of forging new ones [23,24]. The success of nonprofit organizations relies significantly on their capacity to establish high-quality relationships with key stakeholders, including donors, clients, grant makers, seekers, and the broader public [25,26]. The social capital of nonprofits comprises the wealth of resources intricately embedded within these strategic alliances and stakeholder relationships [27]. Xu and Saxton [26] propose that the effective acquisition of social capital, at an elevated level, relies on the scope and quality of stakeholder connections. Their study introduces and demonstrates the significance of 2 primary stakeholder engagement strategies: content-based and connection-based strategies. This study underscores that the attainment of social capital is less about the number of stakeholder engagements and more about the breadth of those engagements. This breadth includes diverse stakeholder connections.

### Social Media–Based Social Capital

Social media–based social capital signifies the diverse social networks that organizations can foster through their engagement on social media platforms [26]. The potential of social media to nurture and sustain web-based–offline social capital is substantial, although its effectiveness varies across platforms and strategies [28]. Platforms such as Facebook (Meta Platforms), Twitter, and Instagram (Meta Platforms) offer distinctive usability features that influence the dynamics of bridging and bonding social capital among their users. An important study conducted by Phua et al [22] examined the impact of 4 major social networking sites (Facebook, Twitter, Instagram, and Snapchat [Snap Inc]) on the development of web-based bridging and bonding social capital among 297 users. Their findings indicate that Twitter users exhibit the highest levels of bridging social capital, followed by Instagram, Facebook, and Snapchat. Conversely, when it comes to bonding social capital, Snapchat users demonstrate the highest levels, followed by Facebook, Instagram, and Twitter. Furthermore, research suggests a direct correlation between the number of followers and the development of bonding social capital [21]. Another study by Xu and Saxton [26], focusing on 198 community foundations, reinforces the importance of social media engagement strategies tailored to multiple intersectoral stakeholders and diverse communication patterns, which substantially contribute to the development of social media–based social capital.

In the context of nonprofit organizations, studies by Henry and Bosman [29] and Lee and Shon [30] underscore the positive impact of web-based social capital generated through social networking sites on charitable outcomes. These studies reveal that the quantity of Twitter followers is linked positively with personal contributions, although not necessarily with full-time equivalent volunteers [30]. Moreover, Xu and Saxton [26], drawing from Twitter data consisting of 198 community foundations, highlight the pivotal role of stakeholder engagement diversity over connection quantities. They emphasize the significance of using multiple communicative cues, such as message elements, and targeting intersectoral and interregional stakeholders in the successful acquisition of social capital through social networking sites. Leveraging social media platforms offers numerous advantages to nonprofit organizations, including the engagement of a donor base within the general population [1,31], the facilitation of communication strategies through the dissemination of information to a broader global audience [32], and the support of advocacy efforts for social change and community mobilization [17]. Svensson et al [33] examined the Twitter use of sport-for-development organizations and identified varying levels of engagement across different entities, potentially limiting the cultivation of social media–based social capital within this sector. Investigating the extent of social media use serves as a valuable tool to inform recommendations aimed at enhancing nonprofits’ web-based presence and fostering social media–based social capital [2,4,34].

These findings underscore the great significance of further investigation into the scope and nature of social media use, particularly within sectors dedicated to service delivery, such as sexual assault programs and organizations, which share a common mission and focus of their efforts. The endeavor to augment social capital through social media within a given sector has the potential to expand the donor and volunteer base, engage the community in matters affecting everyone, and catalyze broader social change at the policy level by mobilizing concerned citizens.

### Aim of the Study

This study aims to address the existing research gap surrounding the use of social media platforms such as Twitter by specific organizations, such as sexual assault support agencies. This study intends to investigate user activities, demographics, and social network structures within these organizations on Twitter. By doing so, we aim to contribute to a better understanding of the current state of social media adoption and social network structures within sexual assault organizations in Canada. In addition, this study provides valuable insights and recommendations for building social capital among the sexual assault organizations on social media. To achieve these goals, we formulated the following research questions (RQs):

- RQ1: What is the demographic profile of sexual assault centers in Canada with official Twitter accounts, including their geographical distribution, years of establishment, and age of the organizations?
  RQ1a: How prevalent are sexual assault centers in Canada with official Twitter accounts, and which provinces and territories have the highest number of centers actively using Twitter?
  RQ1b: What are the geographic locations of sexual assault centers with official Twitter accounts in Canada?
  RQ1c: In what years were the Twitter accounts of sexual assault centers in Canada established, and are there any differences in account creation among provinces and territories?
  RQ1d: What is the average age of these centers since establishing their official Twitter accounts, and do any differences in account creation exist among provinces and territories?
- RQ2: What is the user activity of sexual assault centers in Canada with official Twitter accounts, including their posting frequency by month and year?
  RQ2a: How many sexual assault centers maintain an active Twitter account each year in each province or territory?
  RQ2b: What are the Twitter activity and posting patterns of sexual assault centers while they are active on Twitter?
  RQ2c: How do the Twitter activity and posting patterns vary across provinces and territories?
- RQ3: What are the social network dynamics of bridging and bonding the social capital of sexual assault centers in Canada?
  RQ3a: What are the variations in network size, specifically in terms of followers and followings, among sexual assault centers in different provinces and territories in Canada?
  RQ3b: What is the relationship between followers and followings of these organizations on Twitter?
  RQ3c: What insights can be gained from the social network structure of sexual assault centers on Twitter?

## Methods

### Sampling

This study used purposive sampling to select sexual assault centers in Canada. Our sampling frame was developed by combining lists of sexual assault centers by province and territory from 2 sources: the Canadian Association of Sexual Assault Centres website and the Sexual Assault Centres, Crisis Lines, and Support Services directory. After removing duplicates, our sample frame consisted of 350 sexual assault centers across 10 provinces and 3 territories, providing basic information such as center name, phone number, email, and website. Our inclusion criteria were that the sexual assault center had a Twitter account and had posted at least 1 tweet. To confirm eligibility, a research assistant manually searched the home page of these centers and Twitter pages and conducted Google searches. We determined that 127 organizations had Twitter accounts, but 3 of them had never tweeted anything. As a result, our final sample consisted of 124 Twitter accounts belonging to sexual assault centers across 9 provinces and the Yukon and Northwest Territories. It should be noted that there were no sexual assault centers in Prince Edward Island and Nunavut that used Twitter.

### Twitter Handles’ Acquisition

We collected the Twitter account name, location (eg, Toronto, Ontario), and Twitter handle (eg, @ABCD) for each sexual assault center’s Twitter account. The Twitter handle represented as “@name” is used by followers when replying to, mentioning, and sending direct messages to an account. We identified 22 duplicate Twitter handles among the sampled centers. As a result, our final sampling list consisted of 113 unique Twitter handles obtained from 124 centers. We gathered this information directly from the home page of each sexual assault center’s Twitter account.

### Data Collection

#### Acquisition of Twitter IDs

To collect the data necessary for this study, we obtained Twitter IDs for the 113 unique Twitter handles in our sample. A Twitter ID (eg, 12345678) is a unique numeric value associated with each Twitter handle, and it cannot be changed. We converted each Twitter handle to its corresponding Twitter ID. To ensure the accuracy of our conversions, 2 research assistants verified the results using 3 different websites: TweeterID, CodeOfaNinja, and Comment Picker.

#### Collection of Tweets

We used the 113 Twitter IDs associated with the sampled 124 sexual assault centers to collect their corresponding tweets. To accomplish this, we used Twitter’s academic search application programing interface (API) full archive end point and timeline end point, which allowed us to retrieve tweets published as early as 2006 [35]. We accessed the Twitter API using the native rest API requests. Our data collection process was conducted on March 15, 2023. We downloaded all tweets posted by the sampled centers from the date of each account’s establishment to March 15, 2023. Our data set included 294,350 tweets from 124 sexual assault centers, crisis lines, or support services. We obtained a substantial portion of the total number of tweets published by each Twitter ID on Twitter, specifically, >93.54%.

#### Data Features

We collected several features for each individual tweet message, including the user ID (user_id_str), user account creation date (user_created_at), user location (user_location), username (user_name), user screen name (user_screen_name), tweet creation time (tweet_created_time), full text of the tweet (full_text), and full text of any retweeted status (retweeted_status_full_text).

On Twitter, users commonly use functions such as retweets, replies, mentions, and hashtags. Retweets refer to publicly shared tweets between users and their followers. Users can also add their own comments and media before retweeting. In addition, users can participate in conversations on Twitter by replying to other users and mentioning them in their tweets. Finally, hashtags allowed users to easily follow and search for topics of interest.

### Data Analysis

#### Preprocessing of Raw Data

To address our RQs, we preprocessed the raw data using the following steps:

1. We removed URLs from the tweets.
2. We removed all punctuation marks, with the exception of apostrophes, which are important for contextual meaning in certain words (eg, “We’re”).
3. We removed any bigrams from the set if either of its elements belonged to the list of stop words. For instance, the phrase “The increasing awareness about sexual assault” would generate the bigrams “the increasing,” “increasing awareness,” “awareness about,” “about sexual,” and “sexual assault.” In this case, the stop words “the” and “about” would be removed from the bigrams “the increasing,” “awareness about,” and “about sexual,” leaving the bigrams “increasing awareness” and “sexual assault” in the set.

#### Descriptive Analysis

Descriptive analysis was used to calculate the number of sexual assault centers in each province, the number of centers created each year, and their average age. The age of each sexual assault center was determined by dividing the month of March 2023 by the establishment date of that center. For example, we used R’s *difftime* method (R Core Team) to calculate the age of center A’s Twitter account, which was created on March 12, 2009. By subtracting “2009-03-12” from “2023-03-15” to obtain the time difference, we determined that this center has an age of 14 years.

#### Geolocation Mapping of Sexual Assault Centers

We used the Twitter accounts’ IDs and sexual assault center locations to determine the actual locations of each tweet sent by the 124 centers. The locations were plotted and visualized on a map of Canada. One research assistant manually (QZ) retrieved center location information, including the city, region, and province, from the centers’ official websites and obtained the longitudes and latitudes of the cities where the centers were located. The Google API was used to calibrate the geolocations if the absolute distance discrepancy between manually identified geolocations and Google map geolocations was >6 km. Finally, we developed a Python script to automatically generate D3.js for mapping all the centers with their latitudes and longitudes (the script is available upon request). We used Figma to indicate the cities on the map [36].

#### Social Network Analysis

Social network analysis is one of the most effective techniques for visualizing and assessing network connectivity dynamics, offering insights into patterns of connection and disconnection among participants at a given moment. In our study, we used social network analysis to construct networks from Twitter accounts, where nodes represented accounts and directed edges symbolized follower relationships. We used *Pyvis* [37] and *NetworkX* in Python to create the network, resulting in 111 nodes and 995 edges, which is a visual representation of the relationships within the sexual assault center’s Twitter accounts. To delve deeper, we applied a range of metrics: (1) density, which quantifies the percentage of actual connections within the network; (2) degree centrality, which evaluates a node’s significance by examining its connections, distinguishing between incoming (in-degree) and outgoing (outdegree) connections [38]; (3) eigenvector centrality, which measures a node’s influence in a network by considering the relative scores to connected nodes and is based on the concept that connections to nodes with higher scores exert a more significant influence on determining the node’s score, in contrast to connections with nodes having lower scores [39]; (4) modularity, indicating the network’s community organization strength through clustering [40,41]; (5) betweenness centrality, evaluating an individual’s role as a bridge between unconnected entities, fostering vital connections among clusters, communities, and organizations [38]; and (6) closeness, gauging a node’s centrality in a connected graph by summing the shortest path lengths to all other nodes [42]. These metrics collectively provided a comprehensive understanding of the social network’s structure, shedding light on its various facets and opportunities for relationship cultivation and network analysis.

### Ethical Considerations

The data set and analyses relied on publicly accessible secondary Twitter data; thus, no ethics approval or organizational consent was necessary. The study data presented in this manuscript were subjected to anonymization and deidentification procedures. All personally identifiable information, including but not limited to individual organizations’ identities, pictures, user-specific data, or tweets that have not been rephrased, have been meticulously removed from the data set to ensure complete anonymity.

## Results

### Demographic Profile of Sexual Assault Centers on Twitter

#### Prevalence of Sexual Assault Centers on Twitter

**Figure 1:**
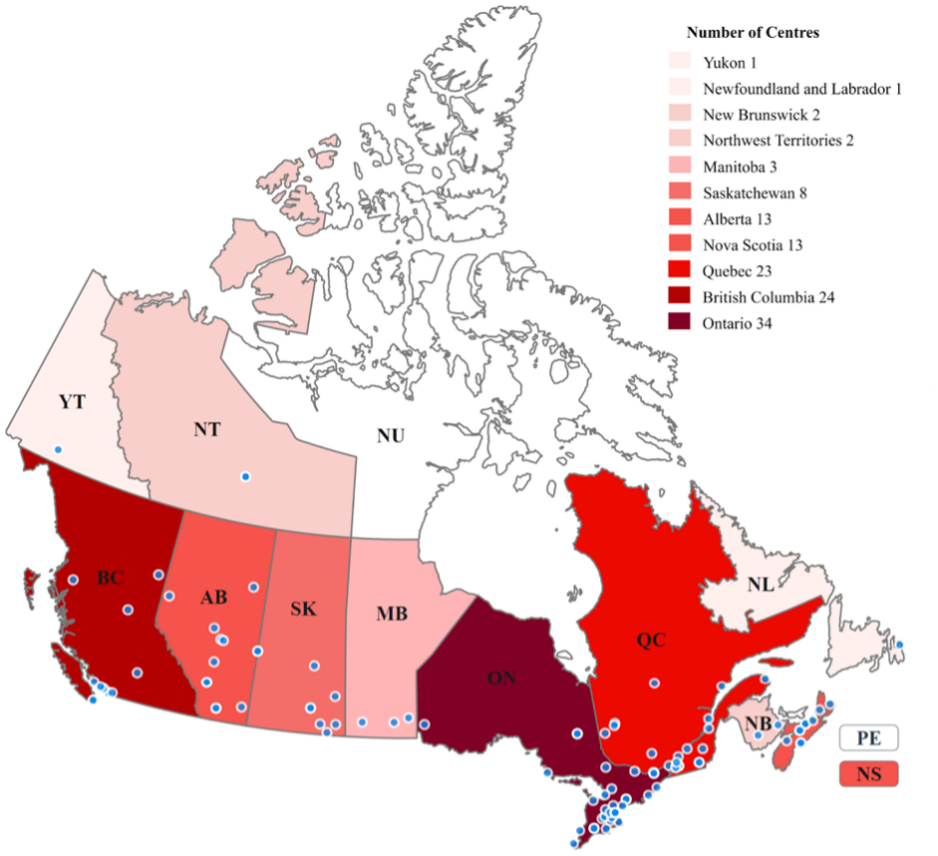
Sexual assault centers in Canada. AB: Alberta; BC: British Columbia; MB: Manitoba; NB: New Brunswick; NL: Newfoundland and Labrador; NS: Nova Scotia; NT: Northwest Territories; NU: Nunavut; ON: Ontario; PE: Prince Edward Island; QC: Quebec; SK: Saskatchewan; YT: Yukon.

We found that 124 (35.4%) centers out of the 350 sampled sexual assault centers have an official Twitter account and have posted at least 1 tweet since their establishment. We investigated the locations of the 124 sexual assault centers in Canada that have official Twitter accounts. The province with the highest number of sexual assault centers was Ontario (n=34), followed by British Columbia (BC; n=24) and Quebec (n=23). These 3 provinces accounted for two-thirds (81/124, 65.3%) of all sampled sexual assault centers in Canada. Newfoundland and Labrador and Yukon have only 1 sexual assault center each. Figure 1 shows the prevalence of sexual assault centers in Canada that have posted tweets.

#### The Geographic Distribution of Sexual Assault Centers on Twitter

To visualize the distribution of sexual assault centers with Twitter accounts, we created geographic distribution maps for Ontario, BC, and Quebec, which had the highest number of centers in our sample. Additional geographic distributions of Twitter accounts in the remaining provinces and territories are presented in Multimedia Appendix 1.

#### Ontario

**Figure 2:**
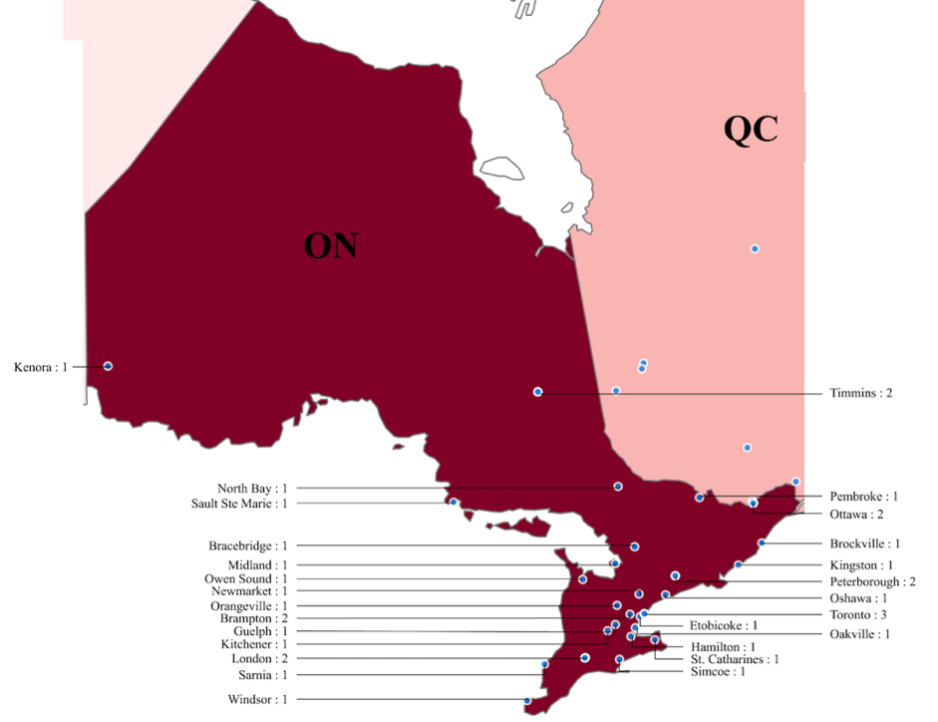
Sexual assault centers in Ontario (ON; 34 centers). QC: Quebec.

Our sample included 34 sexual assault centers from 27 cities in Ontario, shown in Figure 2. Our analysis revealed that most of these centers were concentrated in the southeast region of the province, which is also where most of Ontario’s population resides [43]. Specifically, many centers were found in the cities of Toronto, Ottawa, Peterborough, Timmins, Brampton, and London, which have larger populations in Ontario.

#### British Columbia

**Figure 3:**
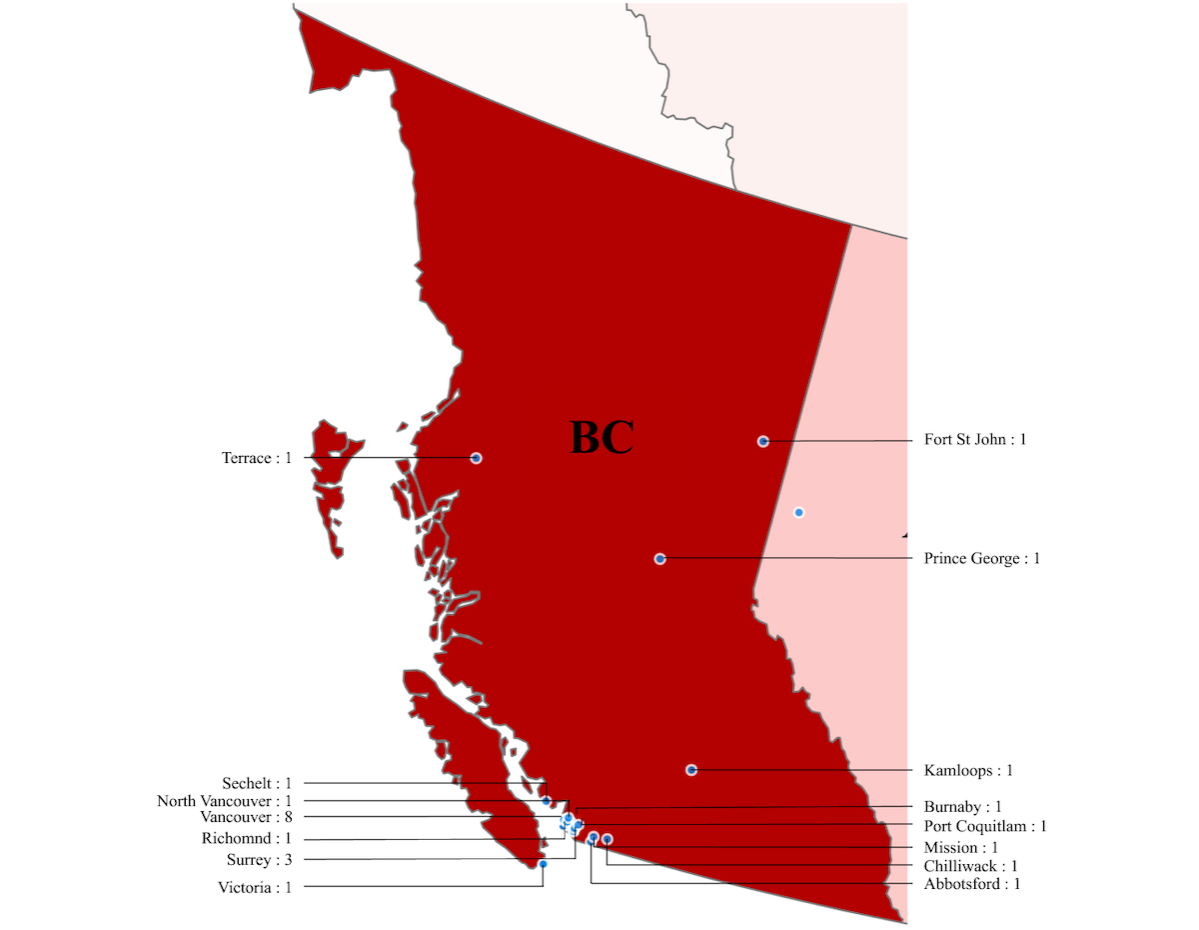
Sexual assault centers in British Columbia (BC: 24 centers).

A total of 24 sexual assault centers were identified in BC, spread across 15 cities, shown in Figure 3. Vancouver had the highest number of centers in the province, followed by Surrey. As per population distribution, most of the population in BC resides in the southern part of the province [44], and similarly, most of the sampled centers are located in the southern region of BC.

#### Quebec

**Figure 4:**
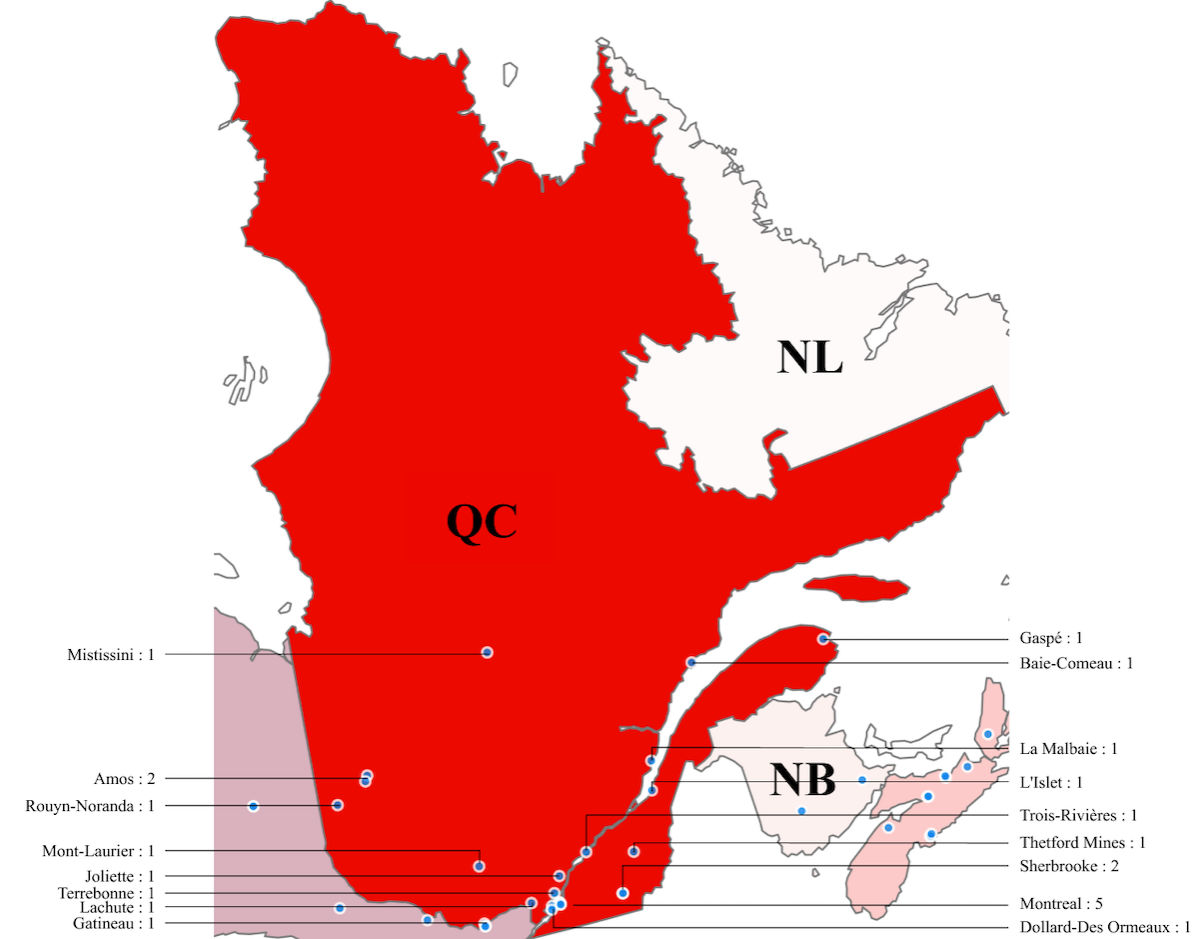
Sexual assault centers in Quebec (QC; 23 centers).

In Quebec, there are 23 sexual assault centers located in 17 cities, with 5 centers located in Montreal, shown in Figure 4. We found that nearly all the centers are situated in the southern region of Quebec, where most of the province’s population resides [45]. Notably, no centers were located in the northern region of the province.

#### Twitter Account Creation Year by Sexual Assault Centers on Twitter: Provincial and Territorial Differences

**Figure 5:**
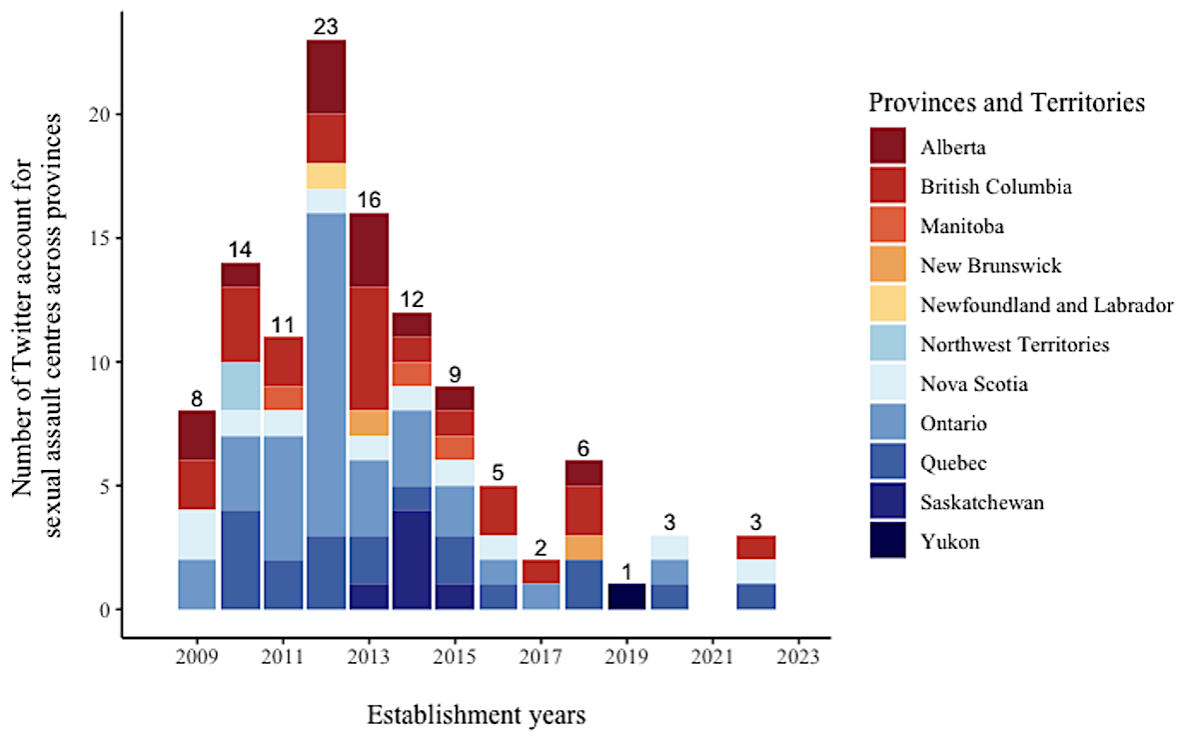
Establishment years of Twitter accounts for sexual assault centers across provinces and territories. The Lloydminster Sexual Assault and Information Centre serves both Alberta and Saskatchewan, so we counted it for both provinces.

We analyzed the year of establishment of the Twitter accounts and assessed whether there were any differences in account creation among provinces and territories. Figure 5 shows our analysis results of the sampled sexual assault centers’ Twitter account creation year. The first Twitter account was created in 2009, and the number of sexual assault centers gradually increased from 2009 to 2012, peaking in 2012 with 23 new accounts. However, from 2013 to 2017, the number of Twitter accounts created by sexual assault centers decreased. In 2019, only 1 sexual assault center created Twitter accounts, and there were no new accounts in 2021. In 2022, we identified 3 centers that had established new Twitter accounts. Notably, all 3 centers that created new Twitter accounts had been in operation for >30 years, as confirmed by our examination of their official profiles.

In addition, we analyzed the distribution of the Twitter accounts created across provinces from 2009 to 2023 and found provincial differences. A total of 8 sexual assault centers from 4 provinces, including Alberta, BC, Nova Scotia, and Ontario, created their Twitter accounts in 2009. From 2009 to 2017, we observed a recurring trend among centers located in Ontario and BC, the 2 provinces with the highest number of centers in Canada, where they established new Twitter accounts on an annual basis. Ontario contributed to the newest Twitter accounts created in 2011 (n=5) and 2012 (n=13). In Quebec, the third-largest province in terms of the number of centers with Twitter accounts, all sexual assault centers began using Twitter after 2010, and remarkably, 7 new centers were created in that year alone. We also noted that most centers in Saskatchewan established their Twitter accounts in 2014. In addition, 3 sexual assault centers located in the Canadian territories also created their Twitter accounts. Specifically, in 2019, one organization in Yukon established a Twitter account, whereas in 2010, two centers located in the Northwest Territories created their Twitter accounts.

#### Average Age of Sexual Assault Centers on Twitter by Province and Territory

We calculated the age of each sexual assault center’s Twitter account since its creation. We determined the duration of time that each sexual assault center had its official Twitter account by subtracting the most recent month of collected tweets (March 2023) from the account creation date. Then, we computed the average length of time for all sexual assault centers in each province and presented the results in Table 1.

**Table 1:** Descriptive statistics for sexual assault centers’ Twitter accounts by province and territory.

Province or territory	Age (y), mean (SD; range)	Portion (%)	Tweets, n	Followers, n	Following, n	Favorites, n	Listed, n
Alberta (n=13)	10.26 (2.39; 5-14)	10.48	39,021	14,115	8167	39,525	296
British Columbia (n=24)	9.33 (3.23; 1-14)	19.35	73,602	29,937	16,635	39,210	730
Manitoba (n=3)	9.30 (1.99; 8-12)	2.42	5691	3004	1421	3966	54
New Brunswick (n=2)	7.31 (3.09; 5-10)	1.61	355	428	480	440	3
Newfoundland and Labrador (n=1)	10.52 (0.00; 11-11)	0.81	1810	1669	504	904	22
Nova Scotia (n=13)	9.55 (4.14; 0-14)	1.61	22,673	14,032	6118	19,016	300
Northwest Territories (n=2)	12.77 (0.23; 13-13)	10.48	1043	659	212	172	34
Ontario (n=34)	10.33 (2.20; 3-14)	27.42	134,467	48,263	27,775	59,601	1111
Quebec (n=23)	9.34 (3.36; 1-12)	18.55	8801	8007	4805	4525	179
Saskatchewan (n=8)	8.75 (0.60; 8-10)	6.45	5899	1826	1915	1859	14
Yukon (n=1)	3.34 (0.00; 3-3)	0.81	1551	187	484	1314	1
Total (n=124)	9.17 (1.29; 0-14)	100	294,913	122,127	68,516	170,532	2744

Table 1 displays the average length of time, SD, and range of the duration of years after the establishment of Twitter accounts by sexual assault centers in each province. For example, 13 sexual assault centers in Alberta created Twitter accounts, and the average number of years since their accounts’ establishment was 10.26 (SD 2.39) years. The range of 5 to 14 indicated that the earliest Twitter account was created in 2009 (March 2023: 14 y), whereas the most recent account was established in 2018 (March 2023: 5 y). These 13 sexual assault centers accounted for 10.5% (n=124) of the total 124 sexual assault centers in Canada.

To obtain a comprehensive understanding of Twitter presence and engagement, we used the metadata of Twitter users related to sexual assault centers in each province, which we obtained from the Twitter timeline API. Specifically, we extracted data such as “followers_count,” “friends_count,” “favorites_count,” and “listed_count” to determine the total number of followers, following, favorites, and listed users, respectively, for each province. We also aggregated the collected data by province to calculate the total number of tweets posted for each province.

### User Activity of Sexual Assault Centers on Twitter

#### Active Twitter Accounts in Canadian Sexual Assault Centers by Province and Territory

**Figure 6:**
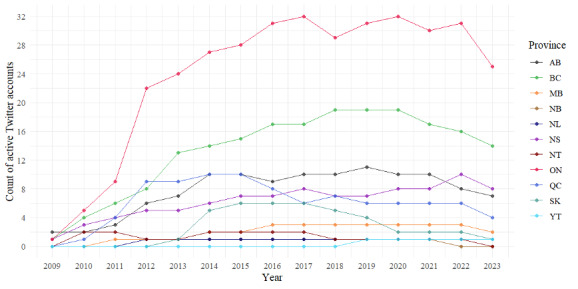
The yearly count of active Twitter accounts maintained by sexual assault centers in each Canadian province or territory (Alberta [AB]: 13 centers; British Columbia [BC]: 24 centers; Manitoba [MB]: 3 centers; New Brunswick [NB]: 2 centers; Newfoundland and Labrador [NL]: 1 center; Nova Scotia [NS]: 2 centers; Northwest Territories [NT]: 2 centers; Nunavut [NU]: 0 centers; Ontario [ON]: 34 centers; Prince Edward Island [PE]: 0 centers; Quebec [QC]: 23 centers; Saskatchewan [SK]: 8 centers; Yukon [YT]: 1 center).

We analyzed the data to determine the number of active Twitter accounts maintained by sexual assault centers in each Canadian province and territory each year. We defined an active account as one that posted at least 1 tweet in a given year. The results indicated a steady increase in the number of active Twitter accounts in Ontario and BC since 2009, as shown in Figure 6. In Quebec, there was an increase in the number of centers from 3 in 2011 to 9 in 2015, but this trend reversed in the following years, indicating that many Twitter accounts became inactive. Although there was an increase in the number of registered Twitter accounts from 13 in 2015 to 16 in 2018, only 4 of these accounts remained active in 2020, down from 9 in 2015. Alberta ranks third or fourth in terms of active Twitter accounts.

In Manitoba, 2 sexual assault centers had active Twitter accounts until 2020. Only 1 center in New Brunswick registered a Twitter account in 2013 but was inactive in 2018 and 2020 while maintaining activity in other years. In Newfoundland and Labrador, the only sexual assault center remained active on Twitter from 2012 to 2020. Nova Scotia consistently showed an increasing trend in the number of active Twitter accounts from 2009 to 2020. Meanwhile, the only center in the Northwest Territories maintained an active Twitter account since 2010. In Saskatchewan, the number of active Twitter accounts increased to 6 in 2015 but decreased to 3 in 2020.

#### Twitter Activity and Posting Patterns of Sexual Assault Centers

**Figure 7:**
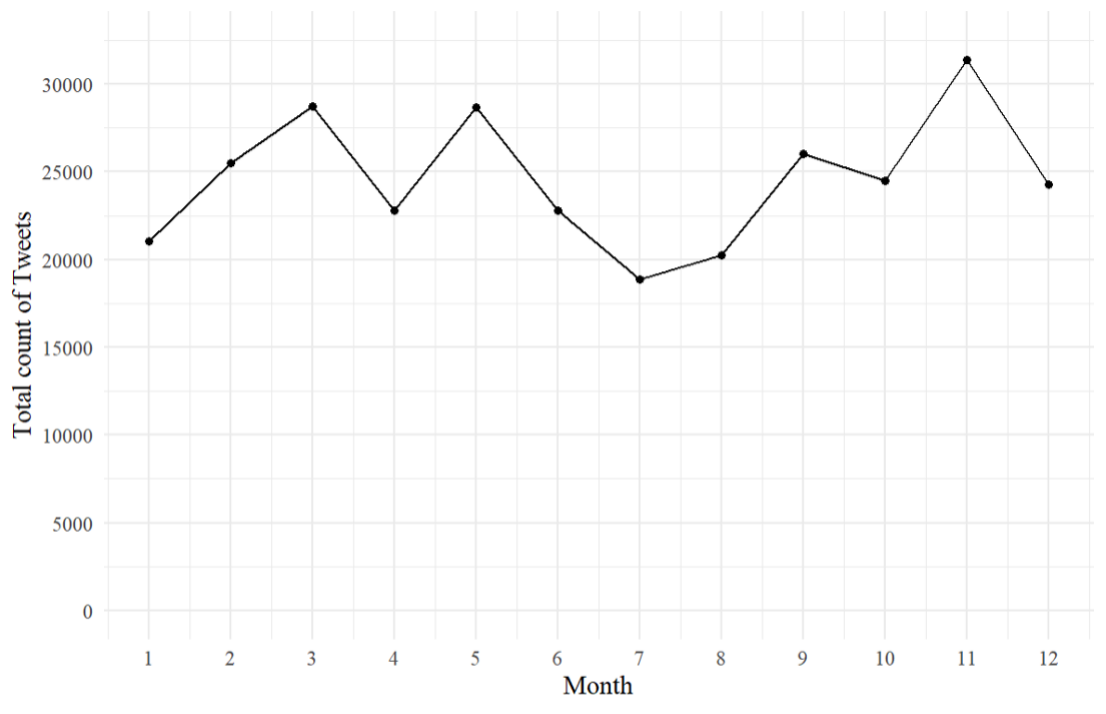
Total count of monthly tweets by all sexual assault centers in Canada.

We also examined the Twitter activity of sexual assault centers in Canada, investigating the popular times for tweeting and the number of tweets posted per month. Figure 7 shows the total number of tweets posted by all sampled centers aggregated by month. Over a 12-year period, these centers posted an average of 12,849 tweets per month. The most active month was November, with a total of 16,239 tweets, whereas the least active was July, with only 9079 tweets. March and May were the peak tweeting months, with >15,000 tweets, whereas August had the fewest tweets, with approximately 9500 tweets.

We analyzed the monthly average tweet count of sexual assault centers during their active status (n=92). We computed the average number of tweets sent by each center per month from the first tweet after creating their account to the last tweet during the data collection period. Results showed that a large majority of the centers have a relatively low tweeting frequency, with the highest frequency of centers (over 20) averaging between 0 to 10 tweets per month. The distribution is right-skewed, showing that as the average monthly tweeting volume increases, the number of centers engaging at the level decreases. A small number of centers tweet between 10 and 30 times per month, and very few centers exceed this range. There are occasional outliers, with one center in particular averaging a significantly higher number of tweets per month, at around 180. This center is an extreme outlier in comparison to the rest of the data set. Overall, our findings suggest that sexual assault centers tend to use Twitter moderately, with the bulk of them tweeting less than 20 times per month, and a very exceptional few tweeting much more frequently.

#### Comparative Analysis of Tweet Activity Across Provinces and Territories

**Figure 8:**
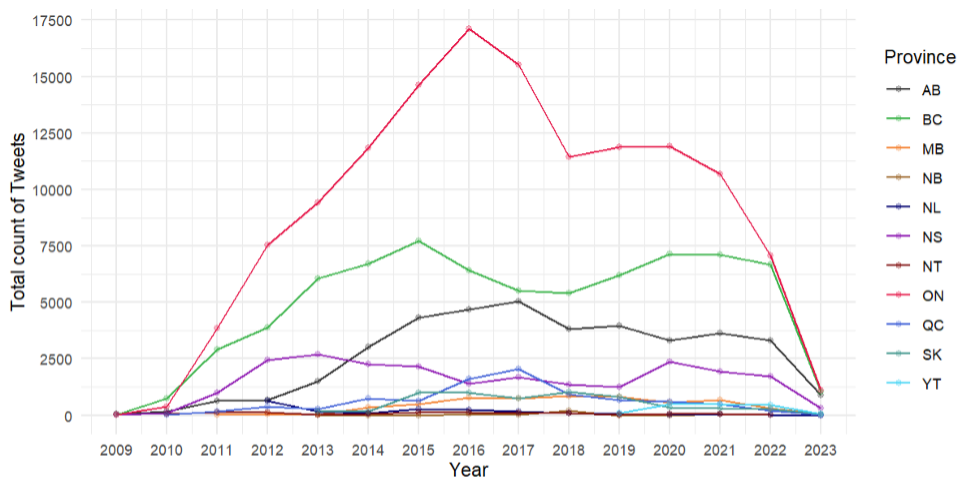
Tweet volume of sexual assault centers by provinces and years. Note: we did not list the NB and NT provinces, which had <100 tweets in the peak month (Alberta [AB]: 13 centers; British Columbia [BC]: 24 centers; Manitoba [MB]: 3 centers; New Brunswick [NB]: 2 centers; Newfoundland and Labrador [NL]: 1 center; Nova Scotia [NS]: 2 centers; Northwest Territories [NT]: 2 centers; Nunavut [NU]: 0 centers; Ontario [ON]: 34 centers; Prince Edward Island [PE]: 0 centers; Quebec [QC]: 23 centers; Saskatchewan [SK]: 8 centers; Yukon [YT]: 1 center).

To answer RQ5, we further analyzed the total number of tweets posted by centers in different provinces and territories each month and compared the number of tweets posted across provinces and territories each year. Figure 8 shows the annual tweet activity generated from all sexual assault center accounts across provinces and territories, with data collected until March 15, 2023. Our analysis of the tweet activity revealed a gradual increase in tweeting volume across provinces and territories until 2017. Notably, Ontario exhibited the highest frequency of tweet activity in 2016, with approximately 17,000 tweets. However, with the exception of accounts in BC and Nova Scotia, the tweet activity gradually declined from 2017 to March 2023, returning to activity levels last observed in 2013 or 2014. It is worth noting that some provinces and territories, such as New Brunswick and Northwest Territories, had <100 tweets in the peak month, and hence, they were not included in our figure. The findings suggest a potential decrease in Twitter activity among sexual assault centers in recent years.

### Social Network Dynamics of Sexual Assault Centers in Canada

#### Network Size

The sexual assault organizations under investigation had an average follower count of 1543 (SD 1555) and an average following count of 819 (SD 876). The range of follower counts was quite diverse, starting at a minimum of 8 followers for one organization and reaching a maximum of 7228 followers for another organization. Similarly, the following counts also showed significant variation, with one center having the lowest count of 1 following, whereas another center had the highest following count of 4458. More detailed information about the Followers, followings, and measurement of social network analysis are in Multimedia Appendix 2.

Sexual assault organizations across 11 provinces exhibited varying average follower counts, ranging from as low as 1 follower in New Brunswick to 17 followers in Ontario, as shown in Table 2. The organization located in Ontario had the highest number of followers, totaling 46. In contrast, some sexual assault organizations in provinces such as Alberta and BC had no followers at all. Similarly, the average number of followings by these organizations across the 11 provinces and territories ranged from 0 in New Brunswick to 18 in Ontario. The organization with the most followings was also situated in Ontario, with a total of 38 followings, whereas several organizations in provinces such as New Brunswick and Quebec had no followings.

**Table 2:** Descriptive statistics of followers and followings from 11 provinces and territories.

Province or territory	Followers, mean (SD; range)	Followings, mean (SD; range)
Alberta	7.55 (6.83; 0-25)	7.91 (6.25; 0-21)
British Columbia	6.48 (8.70; 0-30)	5.52 (5.06; 0-20)
Manitoba	2.67 (2.08; 1-5)	3 (2.65; 1-6)
Manitoba	1 (N/A^a^; 1-1)	0 (N/A; 0-0)
Newfoundland and Labrador	7 (N/A; 7-7)	5 (N/A; 5-5)
Northwest Territories	1.50 (0.71; 1-2)	3 (1.41; 2-4)
Nova Scotia	4.91 (3.86; 0-15)	5.18 (5.95; 0-21)
Ontario	17.20 (11.55; 0-46)	17.60 (10.22; 1-38)
Quebec	4.20 (3.71; 0-13)	4.30 (4.14; 0-14)
Saskatchewan	2 (1.83; 0-4)	1.75 (0.96; 1-3)
Yukon	2.50 (2.12; 1-4)	1.50 (2.12; 0-3)

^a^N/A: not applicable.

#### Relationship Between Followers and Followings on Twitter

**Figure 9:**
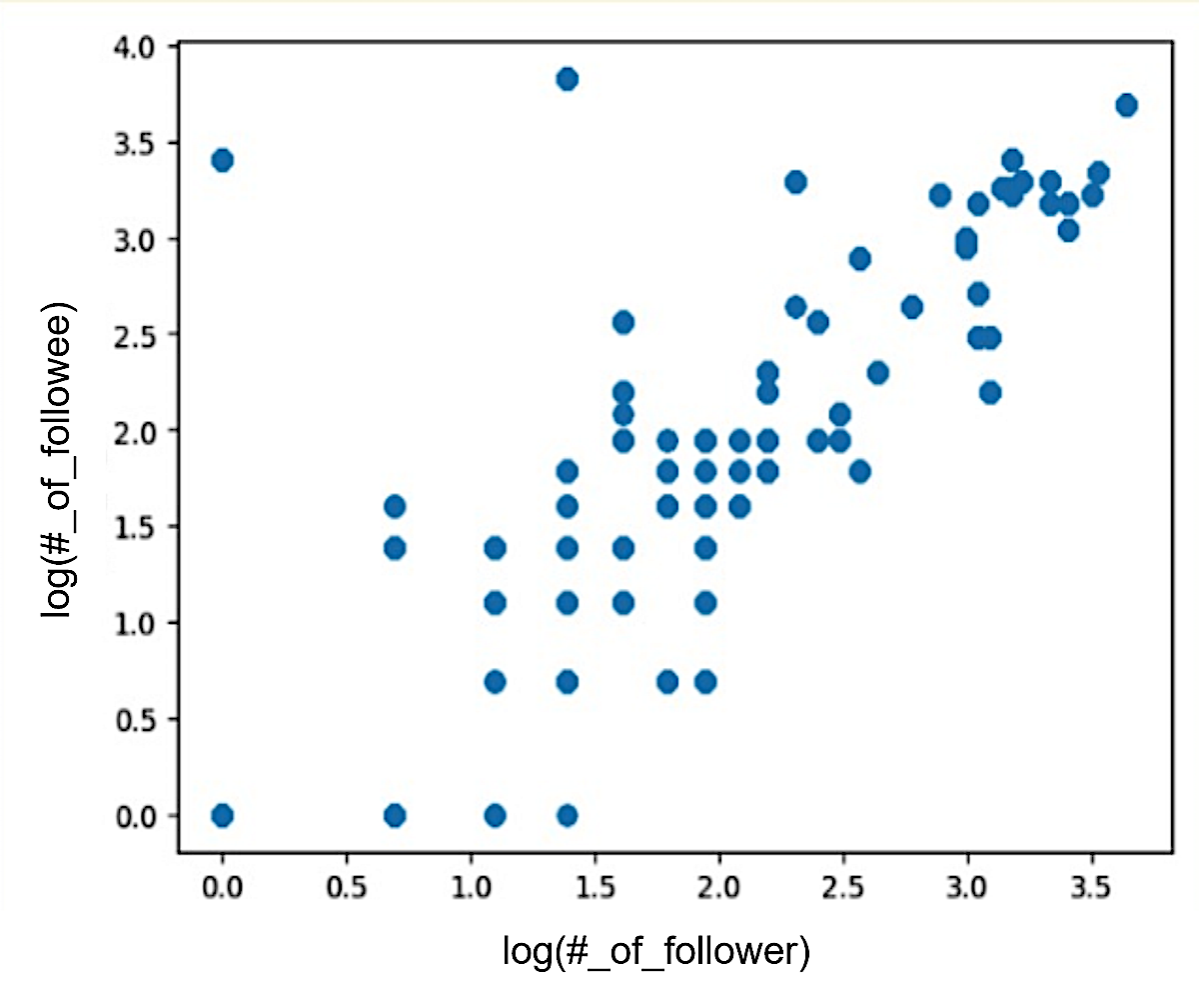
Relationship between followers and followings on Twitter.

We analyzed the Twitter user network by exploring the connections between the followers and the following lists. Figure 9 shows the log-log plot of the correlation between followers and followings. Each point on the graph represents an individual user, with the x-axis representing the user’s followers and the y-axis indicating their following count. The plot demonstrates that as the number of followers continued to increase, the followings also indicated an increasing trend. At the middle of the plot, users with a medium number of followers have a high number of followings.

#### Analysis of Twitter Social Network Structure and Node Categorization

**Figure 10:**
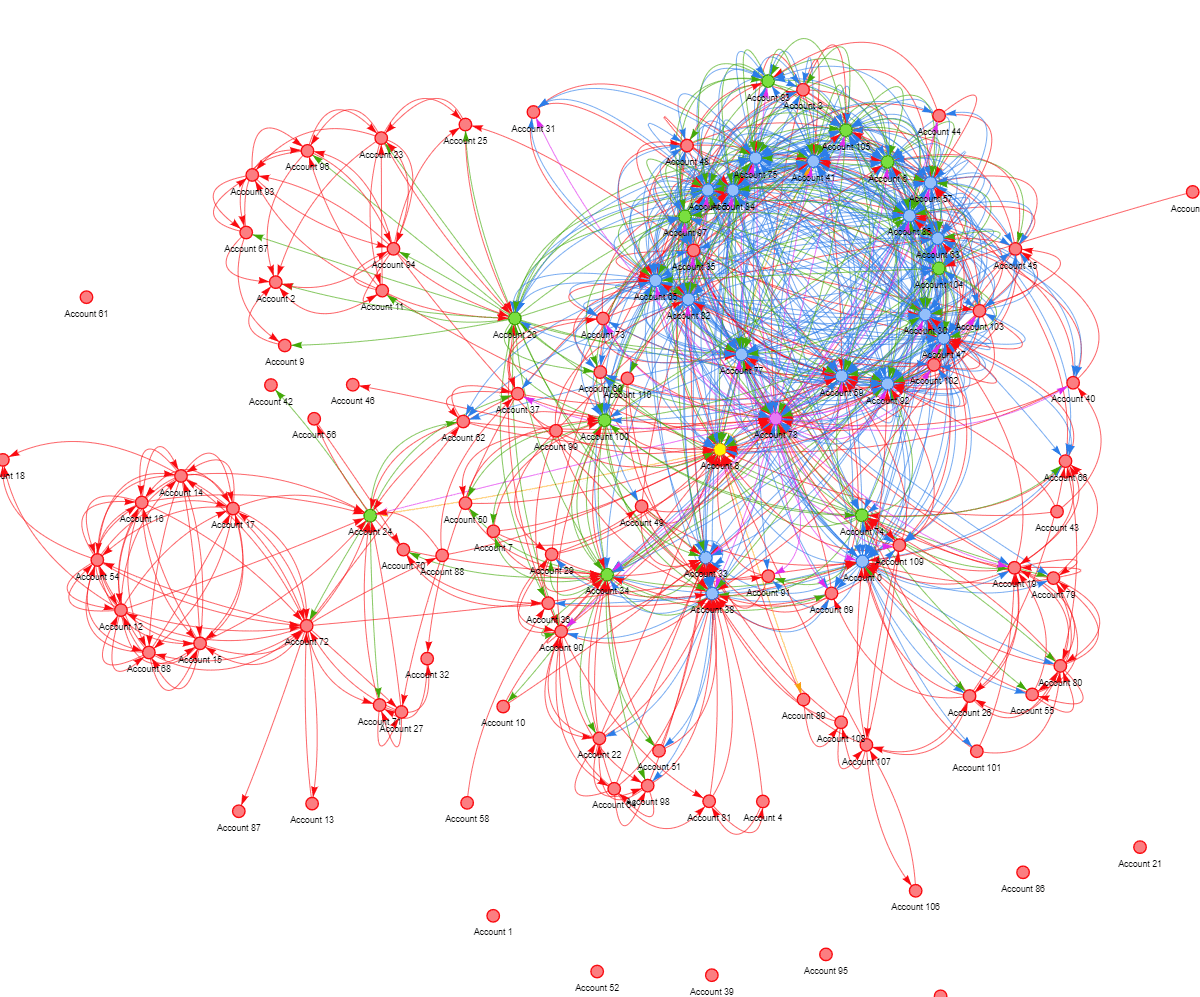
Social network structure.

Figure 10 presents a full network map, illustrating the relationships between followers and followings among 111 sexual assault centers on Twitter. This graph features 111 nodes and 995 edges. Each node represented a sexual assault center on Twitter, and each edge is directional, with arrows symbolizing “x follows y” relationship. In this context, Y serves as the following node, implying that it is followed by other nodes, whereas X acts as the follower node, signifying that it follows other nodes.

Among the 111 sexual assault centers on Twitter, the average count of both followers and followings was approximately 9. There is a notable variability in these counts, with a SD of 9.15 for followings and 9.85 for followers. The maximum number of followings observed was 38, whereas the maximum number of followers reached was 46, with a minimum count of 0.

We categorized them into different categories to account for the variability in the number of followers. The graph illustrates nodes of various colors, each representing a specific range of followers. Red denotes those with <11 followers, green indicates 11 to 21 followers, blue represents 21 to 31 followers, purple signifies 31 to 41 followers, and yellow indicates those with ≥41 followers. Notably, there is a single green node, which stands out with 46 followers. The in-degree centrality is 0.42, which is the highest value among all the nodes, underscoring their significance. In addition, they possess a closeness score of 0.49, ranking them within the top 1% among all nodes, implying their high level of proximity to other nodes and less dependency on others for information transmission. Furthermore, their betweenness score was 0.076, signifying their involvement in a substantial number of shortest paths and positioning them among the top 7 nodes in the betweenness ranking. We classify the sexual assault centers that meet the criteria of high closeness, in-degree centrality, low betweenness, and >41 followers and followings as *social queens*.

The figure also draws attention to a cluster of sexual assault centers characterized by a smaller number of followers and followings. We classified these nodes as marginalized entities. These centers have a limited impact on information transmission on Twitter, leading to their closeness and other metrics registering at 0.

#### Identification of Modular Patterns and Key Nodes in Twitter Network

**Figure 11:**
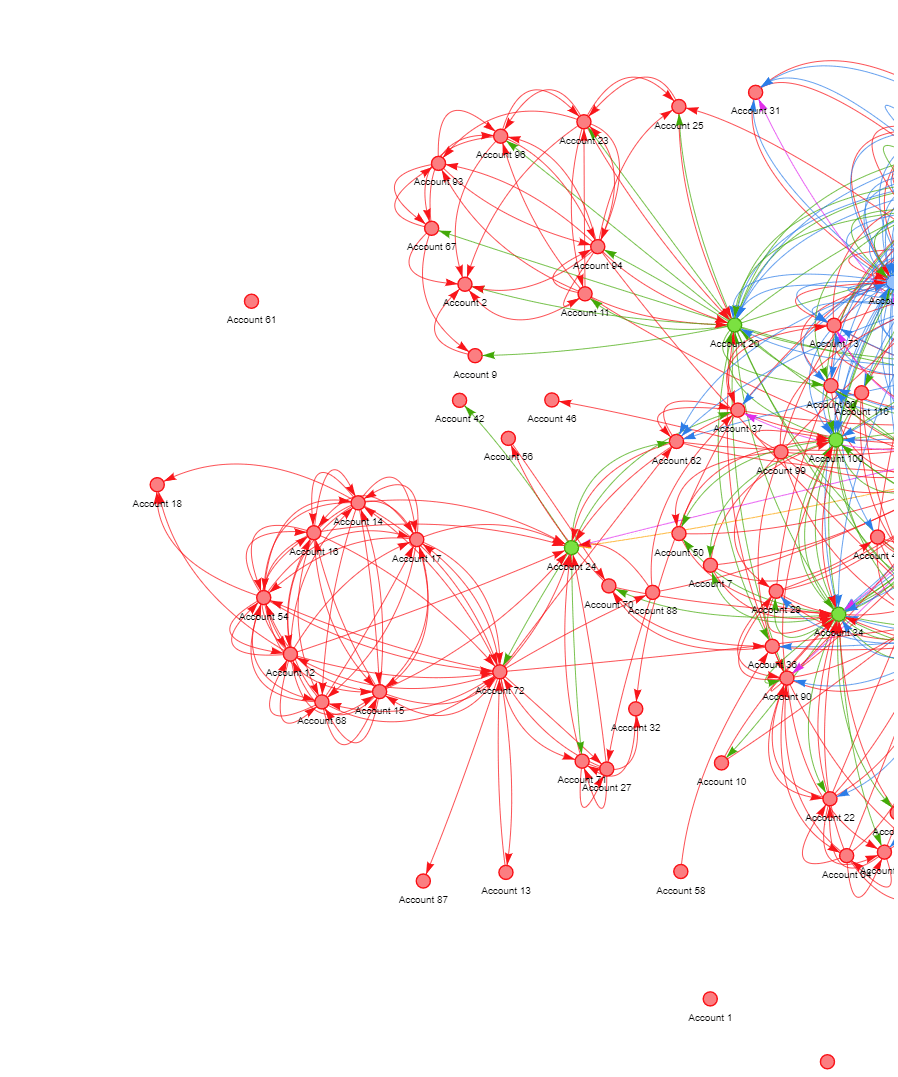
Identification of modular patterns and key nodes in Twitter network.

Figure 11 is a section from Figure 10, in which distinct patterns emerge as certain nodes cluster together into modularities. As an example, we can examine a particular section of Figure 10 where a modularity forms—a small cluster consisting of several nodes that mutually follow each other. We incorporated eigenvector centrality to interpret this modularity. Within the bottom-right corner of this modularity, we find that accounts 24 and 72 (anonymous Twitter handle_names) exhibit relatively high values of eigenvector centrality. A higher eigenvector centrality score implies greater significance of the node when compared with its neighboring points. Furthermore, the importance of the node itself is directly linked to the significance of the neighboring nodes connected to it. Consequently, this specific node can be regarded as the “social queen” within this particular modularity.

## Discussion

### Principal Findings

This study represents a pioneering effort to conduct a comprehensive analysis of Twitter’s social network, user activities, and demographics within the context of sexual assault centers in Canada. By mapping and analyzing their Twitter practices, this research contributes to a better understanding of the social media landscape of sexual assault support organizations in Canada. The findings underscore the potential of Twitter as a platform for sexual assault organizations to build social capital, enhance their influence, and expand their reach. Moreover, it highlights the need for tailored engagement strategies that consider regional disparities and the unique characteristics of each province and territory. Our findings align with the broader literature on social capital, specifically bridging and bonding social capital. Among the various social media platforms, Twitter emerges as a valuable data set to study sexual violence [46] as well as a notable facilitator of bridging social capital, consistent with previous research that has underscored Twitter’s ability to connect organizations with a diverse and expansive audience [22].

### Demographic Profile of Sexual Assault Centers on Twitter

The results of this study reveal a substantial presence of sexual assault centers in Canada on Twitter, signifying their acknowledgment of Twitter as a valuable communication and social capital development tool. Out of the 350 sampled centers, 124 (35.4%) maintain an active Twitter presence, highlighting the significant proportion of sexual assault organizations in Canada that recognize Twitter’s efficacy as a communication medium for engaging with their stakeholders and the public. This trend aligns with the broader nonprofit sector, where most large and mid-sized nonprofits maintain at least 1 social media account [9]. However, it is worth noting that effective communication on Twitter may be hindered by a reliance on broadcasting rather than engaging in dialogue, as observed in previous research [47]. The success of acquiring social capital through social media appears to depend on the extent and quality of stakeholder connections, emphasizing the importance of diverse engagement strategies and the diversity and complexity of message elements [26]. In the context of sexual assault centers and support services, social media–based social capital holds significant potential for increasing the donor and volunteer base, engaging with the community on social issues, and promoting wider social change [33].

The geographic distribution of sexual assault centers with Twitter accounts highlights regional variations in Twitter use and engagement. Ontario, BC, and Quebec emerged as the provinces with the highest number of centers using Twitter, collectively accounting for two-thirds of all sampled centers in Canada, indicating higher levels of social capital in those regions owing to increased opportunities for information sharing, emotional kinship, trust, and social support [20]. The concentration of centers in these provinces aligns with their higher population densities and emphasizes the importance of social media platforms, such as Twitter, in reaching a broader audience consistently [43]. The southeast regions of Ontario and Quebec as well as the southern region of BC showed a higher concentration of sexual assault centers with Twitter accounts, likely reflecting the higher population densities in these areas. Furthermore, spatial distribution may influence the topics and issues addressed in their tweets. In BC, the distribution of sexual assault centers was also concentrated in specific regions, such as Vancouver. The content of tweets from centers in these regions may reflect local concerns and initiatives. It is essential to consider the unique characteristics and needs of each region when developing communication strategies and leveraging social media platforms for social capital enhancement.

The average age of sexual assault centers’ Twitter accounts was calculated to determine the duration of their presence on the platform. The findings showed that the average duration varied across provinces and territories, ranging from 5 to 14 years. The Northwest Territories had the longest average duration of 12.77 years, indicating a relatively early adoption and subsequent use of Twitter among sexual assault centers in the territory. In contrast, provinces or territories such as Yukon and New Brunswick had a shorter average duration. These variations in account age reflect differences in the timing of adoption and highlight the diverse trajectories of Twitter use among sexual assault centers across Canada. These differences may be influenced by organizational factors, regional context, or resource availability. Centers with older Twitter accounts may have accumulated more followers and established stronger web-based communities, whereas newer accounts may need to focus on building and expanding their web-based presence.

### User Activity of Sexual Assault Centers on Twitter

Patterns of account creation offer insights into the temporal dynamics of engagement and emphasize the need for continuous and consistent communication efforts. The recent decline in the creation of new Twitter accounts by sexual assault centers in recent years may signal a saturation point, where most centers have already established their Twitter presence. Alternatively, it could be attributed to factors such as resource constraints, changing organizational priorities, limited staff dedicated to communication practices, or a shift in focus to other social media platforms.

The examination of the average monthly tweet count for active sexual assault centers provides valuable insights into their Twitter activity levels. The results indicate variations in tweet frequency across provinces, with Ontario and BC consistently demonstrating higher tweet volumes compared with other provinces. This observation aligns with the higher number of active Twitter accounts and underscores the importance of ongoing engagement and dialogue with stakeholders through regular tweets.

### Social Network Dynamics of Sexual Assault Centers in Canada

The findings related to social network dynamics reveal the landscape of Twitter engagement among sexual assault organizations. On average, these organizations have amassed approximately 1543 followers, demonstrating their capacity to reach a substantial audience. Simultaneously, they follow an average of 819 other accounts, indicating their active involvement within the Twitter community. This indicates the potential for these organizations to disseminate information, provide support, and raise awareness about their critical missions. This study aligns with the perspective emphasizing the importance of bridging social capital facilitated by Twitter’s ability to connect with various stakeholders, including service recipients, donors, and the general public [26]. It highlights the potential and disparities in bridging social capital among sexual assault centers across provinces and territories in Canada, suggesting that these organizations can better leverage Twitter to establish connections beyond their immediate constituencies. This aligns with the notion that social media platforms such as Twitter can extend an organization’s reach and promote the flow of information across various stakeholders [22].

The findings also uncovered regional disparities in Twitter engagement among sexual assault organizations in Canada. Sexual assault organizations across Canada’s provinces exhibited varying degrees of Twitter activity. Although some provinces, such as Ontario, displayed robust engagement, others, such as New Brunswick, had limited presence and following. Our observation resonates with previous studies (eg, [9]) that have emphasized the role of regional context in shaping nonprofit organizations’ social media use. These regional disparities suggest the need for tailored strategies to maximize the impact of Twitter engagement, considering the unique characteristics and needs of each province.

Within the context of nonprofit organizations, research has indicated a positive relationship between follower count and bonding social capital [21]. Our study aligns with this perspective by demonstrating a positive association between followers and followings. As follower counts increase, there is a corresponding increase in followings, indicating a proactive approach by organizations to engage with their audience. This observation underscores the importance of reciprocity and interaction on Twitter. This suggests that a larger number of followers on Twitter can contribute to increased financial support, in line with the positive impact of web-based social capital generated through social networking sites on charitable outcomes [29,30].

Within the intricate network of sexual assault centers on Twitter, we identified nodes with distinct characteristics. Some organizations emerged as “social queens,” characterized by high in-degree centrality, closeness scores, and low betweenness, coupled with substantial followers and followings. These “social queens” play pivotal roles in information transmission, networking, and community building. This finding suggests that organizations can strategically use Twitter to enhance their influence and reach within their fields of operation. However, this study also highlights the presence of marginalized entities with limited follower counts, which may face challenges in impacting information transmission on Twitter. This underscores the importance of proactive engagement strategies for organizations seeking to maximize their impact through social media. Sexual assault organizations can benefit from a comprehensive understanding of their social network structures, enabling them to identify opportunities to strengthen their social capital, expand their donor base, and effectively engage the community.

### Limitations

This study had some limitations. First, the findings are specific to sexual assault centers in Canada and may not be applicable to other countries or regions owing to cultural, social, and organizational differences. Each country or region may have unique characteristics that influence the use of Twitter and other social media platforms by sexual assault organizations. Second, the study focuses solely on Twitter data and does not consider the use of other platforms such as Facebook, Instagram, or Snapchat, which could provide additional insights into communication strategies and social media practices. Therefore, the findings of this study may not provide a comprehensive understanding of the organizations’ overall social media use. Third, the study is cross-sectional, providing a snapshot of Twitter use at a specific time, and does not capture longitudinal changes or trends. A longitudinal study would offer more detailed insights into the evolution of Twitter practices and the effectiveness of communication strategies used by these organizations. For example, we lack information about growth and changes in the number of followers over time. The follower counts remained static at the time of data collection. Future studies may need to explore how follower counts evolve dynamically to gain deeper insights. Fourth, the study primarily focused on describing Twitter use at the organizational level rather than evaluating the effectiveness or outcomes of the communication strategies used. Further research is required to assess the impact and outcomes of social media use in this context. Fifth, this study did not have access to demographic information related to the organizational size of the nonprofits, which typically includes factors such as the number of employees, volunteers, or annual budget. Unfortunately, Twitter does not provide access to such data, resulting in its absence from this study. Finally, the study does not delve deeper into the content of tweets posted by sexual assault organizations on Twitter. Future studies could explore the thematic analysis of tweets, sentiment analysis to understand the emotional tone of their messages, and the effectiveness of specific content strategies used by these organizations to engage their audience and advocate for their cause.

### Conclusions

In conclusion, this study provides valuable insights into the current use and social structure of Twitter by sexual assault centers, crisis lines, and support services in Canada. The findings highlight the widespread adoption of Twitter among these organizations and the potential for leveraging social media platforms to build social capital. By recognizing regional disparities, identifying key players, and understanding the dynamics of followers and followings, sexual assault organizations can better navigate the Twitter landscape to further their missions of promoting awareness and support for survivors of sexual assault. Further research in this area can explore the long-term impact of social media use on organizational outcomes and stakeholder perceptions into enhancing social capital within the nonprofit sector and beyond, providing additional guidance for effective communication practices in the nonprofit human services sector and ultimately contributing to the broader goals of these organizations.

## Appendix

Multimedia Appendix 1 Sexual assault centers with official Twitter accounts in Alberta, New Brunswick, Newfoundland and Labrador, Nova Scotia, Manitoba, Saskatchewan, Northwest Territories, and Yukon.

Multimedia Appendix 2 Followers, followings, and measurement of social network analysis.

## Abbreviations

API: application programing interface
BC: British Columbia
RQ: research question
