# Supplementary material for: Bridging and Bonding Social Capital by Analyzing the Demographics, User Activities, and Social Network Dynamics of Sexual Assault Centers on Twitter: Mixed Methods Study
Source: J Med Internet Res. 2024 Mar 27;26:e50552. doi: 10.2196/50552 (PMC11007606; doi:10.2196/50552)

**Appendix**

**Figures: Sexual assault centres with official Twitter accounts in Alberta (AB), New Brunswick (NB), Newfoundland and Labrador (NL), Nova Scotia (NS), Manitoba (MB), Saskatchewan (SK), Northwest Territories (NT) and Yukon (YT).**


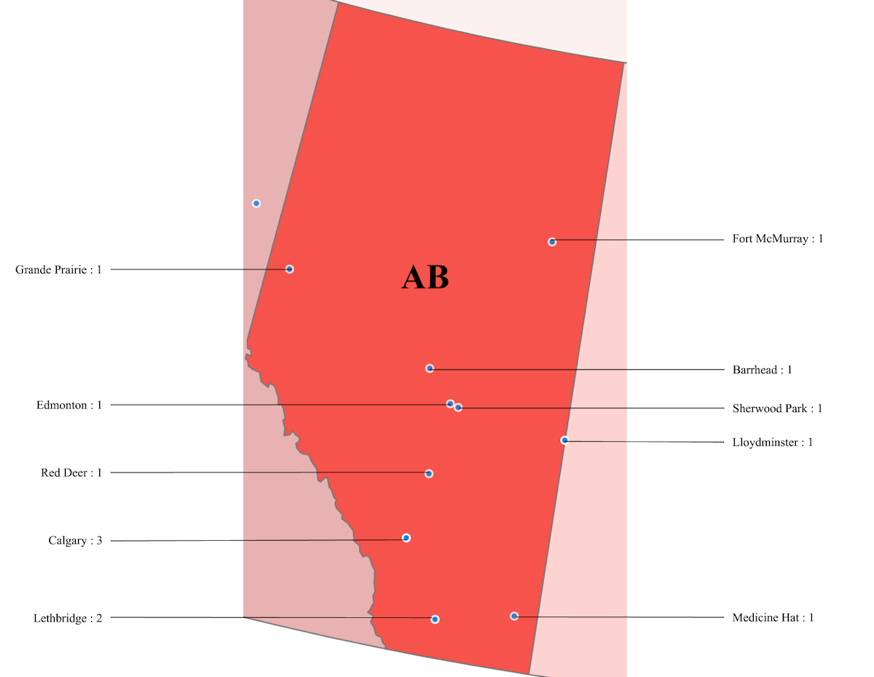

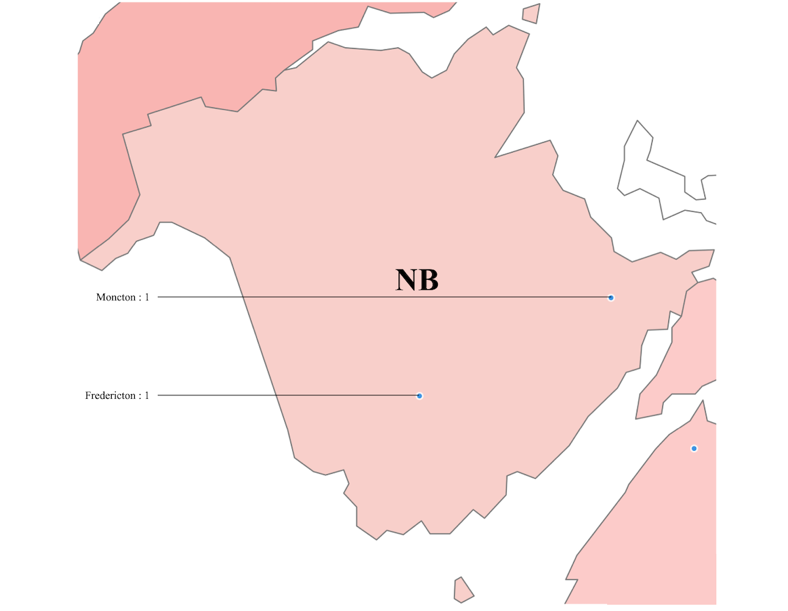

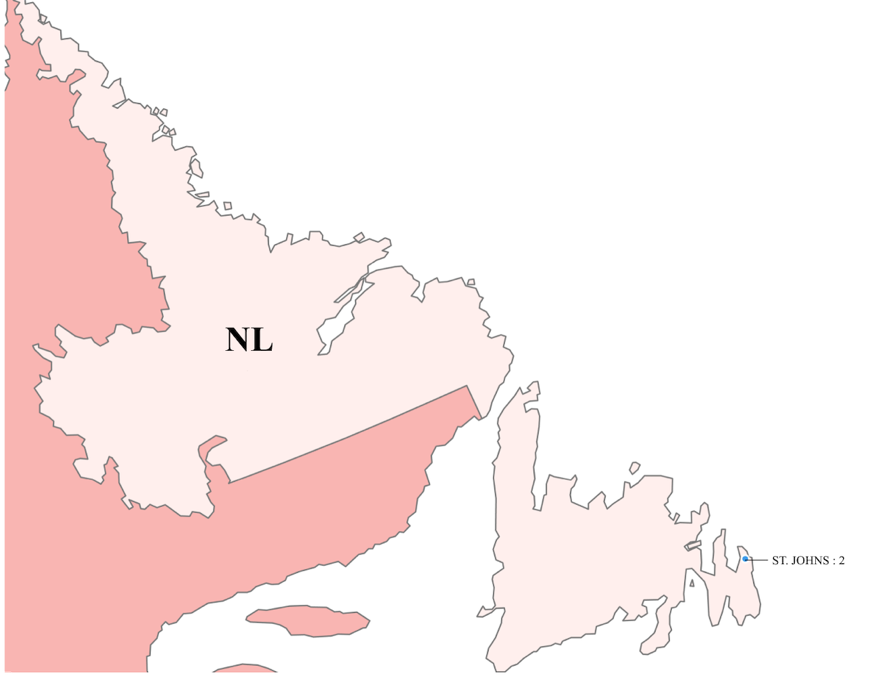

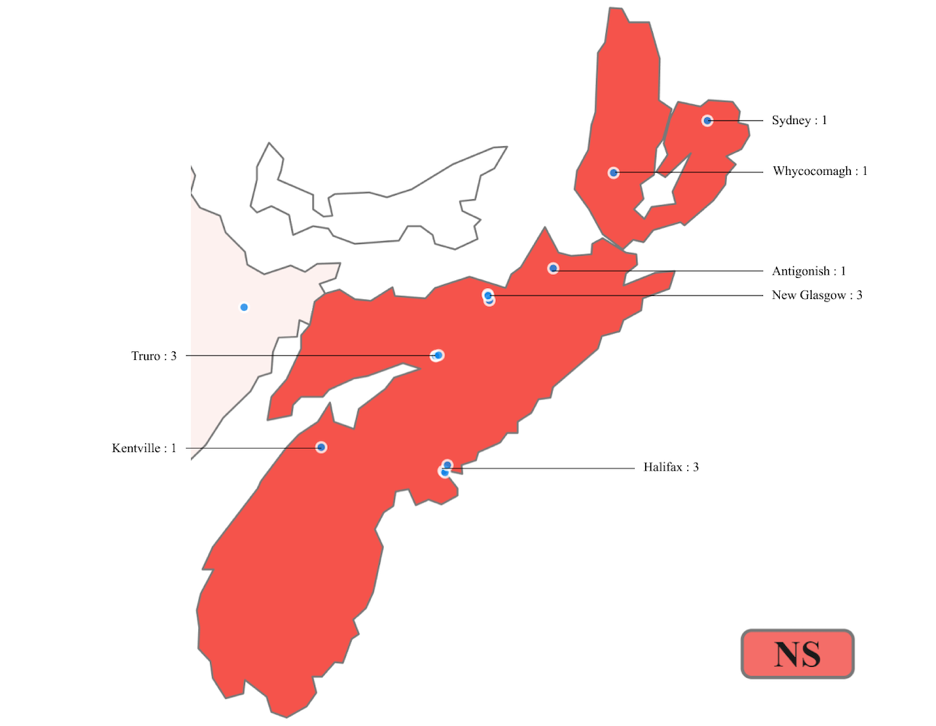


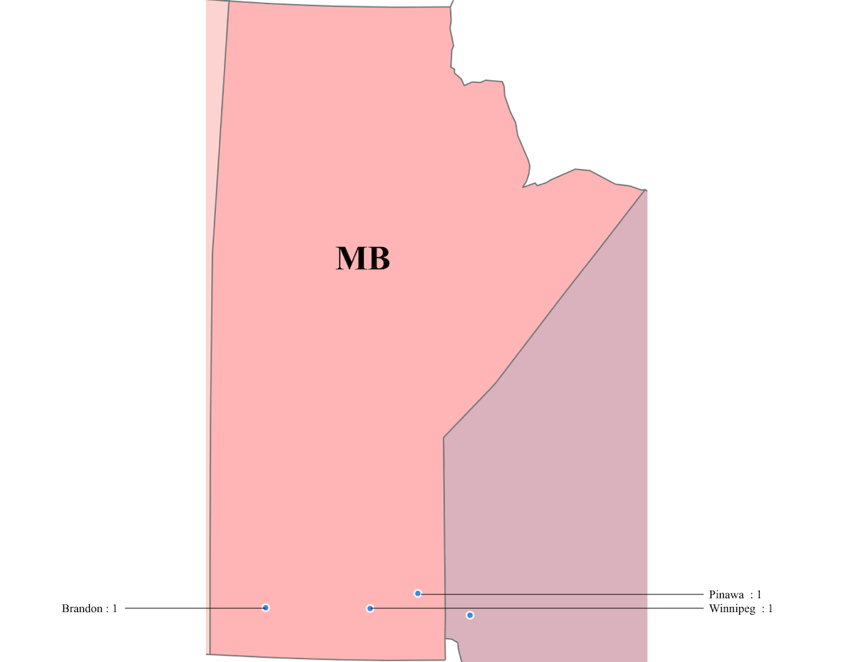


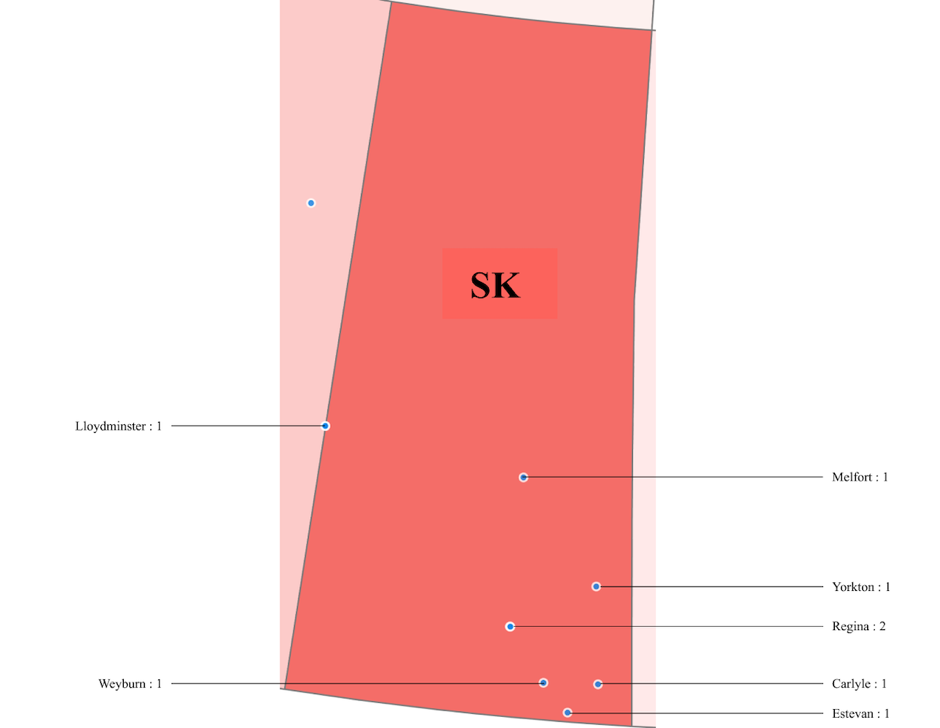


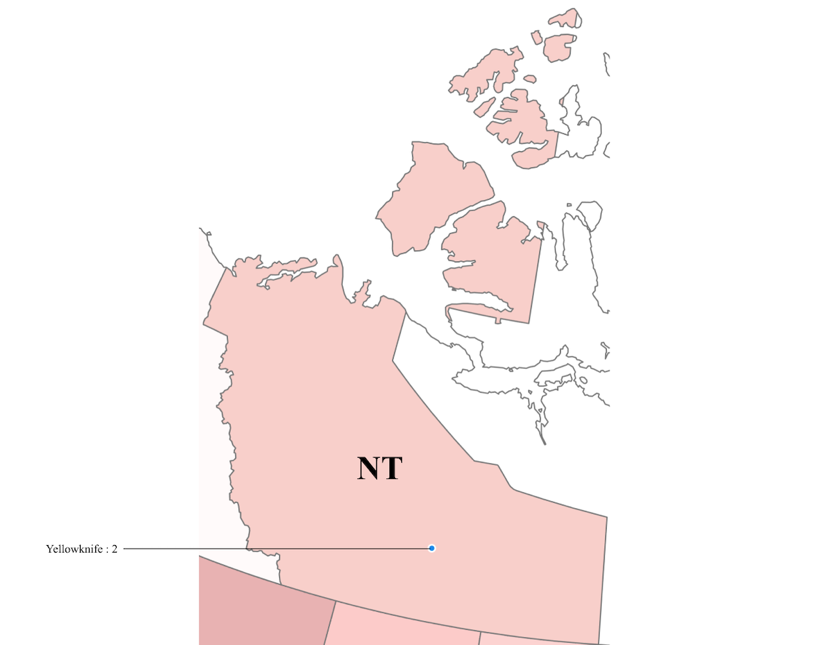

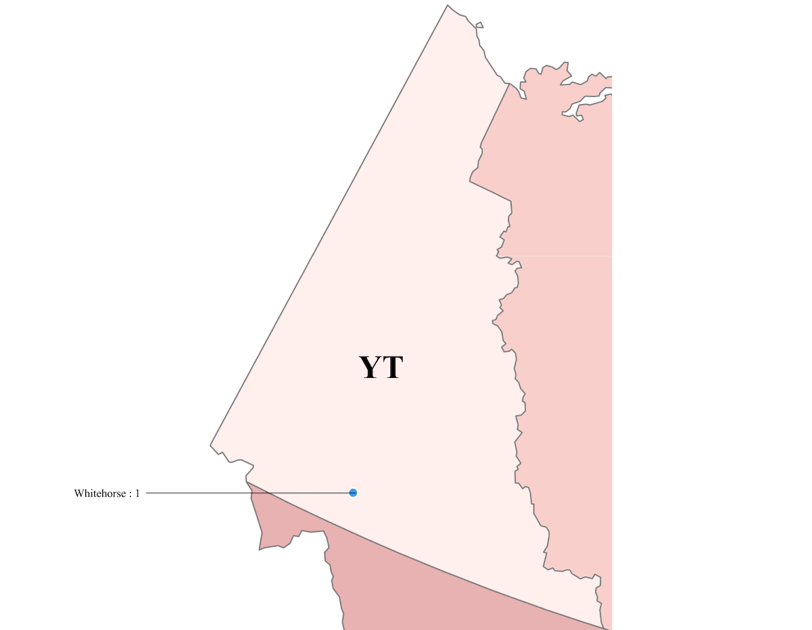

Supplement: Multimedia Appendix 1 [file jmir_v26i1e50552_app1.docx]
