# Supplementary material for: Bridging and Bonding Social Capital by Analyzing the Demographics, User Activities, and Social Network Dynamics of Sexual Assault Centers on Twitter: Mixed Methods Study
Source: J Med Internet Res. 2024 Mar 27;26:e50552. doi: 10.2196/50552 (PMC11007606; doi:10.2196/50552)
Supplement: Multimedia Appendix 2 [file jmir_v26i1e50552_app2.docx]

**Appendix Followers, Followings, and Measurement of SNA**

| **Twitter handle** | **Nodes** | **Follower** | **Following** | **In-degree**  **Centrality** | **Out-degree Centrality** | **Eigenvector**  **Centrality** | **Betweenness** | **Closeness** |
| --- | --- | --- | --- | --- | --- | --- | --- | --- |
| aasasmembership | 0 | 25 | 18 | 0.2273 | 0.1636 | 0.1483 | 0.1214 | 0.4319 |
| AbVictim | 1 | 0 | 0 | 0.0000 | 0.0000 | 0.0000 | 0.0000 | 0.0000 |
| adsumforwomen | 2 | 7 | 0 | 0.0636 | 0.0000 | 0.0059 | 0.0000 | 0.2651 |
| AmeliaRising | 3 | 7 | 11 | 0.0636 | 0.1000 | 0.0681 | 0.0000 | 0.3113 |
| AnnDavisSociety | 4 | 1 | 2 | 0.0091 | 0.0182 | 0.0001 | 0.0000 | 0.1950 |
| AnovaFuture | 5 | 24 | 28 | 0.2182 | 0.2545 | 0.2010 | 0.0140 | 0.3702 |
| ARCCLG | 6 | 18 | 13 | 0.1636 | 0.1182 | 0.1626 | 0.0010 | 0.3433 |
| AVP_UVic | 7 | 2 | 4 | 0.0182 | 0.0364 | 0.0089 | 0.0007 | 0.2828 |
| AWHL | 8 | 46 | 4 | 0.4182 | 0.0364 | 0.2597 | 0.0760 | 0.4868 |
| awrcsasa_ | 9 | 2 | 1 | 0.0182 | 0.0091 | 0.0046 | 0.0000 | 0.2572 |
| bcmalesurvivors | 10 | 1 | 2 | 0.0091 | 0.0182 | 0.0048 | 0.0000 | 0.2751 |
| BryonyHouse | 11 | 3 | 7 | 0.0273 | 0.0636 | 0.0049 | 0.0056 | 0.2534 |
| calacsabitibi | 12 | 6 | 9 | 0.0545 | 0.0818 | 0.0001 | 0.0039 | 0.2125 |
| CalacsBC | 13 | 1 | 1 | 0.0091 | 0.0091 | 0.0001 | 0.0000 | 0.2097 |
| calacsch | 14 | 6 | 9 | 0.0545 | 0.0818 | 0.0001 | 0.0038 | 0.2125 |
| CALACSdeGranby | 15 | 7 | 8 | 0.0636 | 0.0727 | 0.0001 | 0.0014 | 0.2131 |
| CALACSdelOuest | 16 | 6 | 8 | 0.0545 | 0.0727 | 0.0000 | 0.0001 | 0.1727 |
| CalacsEstrie | 17 | 7 | 7 | 0.0636 | 0.0636 | 0.0001 | 0.0013 | 0.2131 |
| CalacsGaspesie | 18 | 3 | 0 | 0.0273 | 0.0000 | 0.0000 | 0.0000 | 0.1746 |
| CASASC2 | 19 | 10 | 9 | 0.0909 | 0.0818 | 0.0421 | 0.0089 | 0.3477 |
| CentreAvalon | 20 | 15 | 21 | 0.1364 | 0.1909 | 0.0847 | 0.1023 | 0.3508 |
| ChezDorisRefuge | 21 | 0 | 0 | 0.0000 | 0.0000 | 0.0000 | 0.0000 | 0.0000 |
| chimoservices | 22 | 5 | 6 | 0.0455 | 0.0545 | 0.0133 | 0.0021 | 0.3054 |
| ChrysalisHouseA | 23 | 5 | 7 | 0.0455 | 0.0636 | 0.0056 | 0.0068 | 0.2566 |
| Citad_elle | 24 | 13 | 5 | 0.1182 | 0.0455 | 0.0295 | 0.1265 | 0.3843 |
| CSAC16 | 25 | 4 | 2 | 0.0364 | 0.0182 | 0.0096 | 0.0004 | 0.2634 |
| CSACLeth | 26 | 5 | 7 | 0.0455 | 0.0636 | 0.0147 | 0.0064 | 0.3009 |
| CVASM_MSAC | 27 | 3 | 3 | 0.0273 | 0.0273 | 0.0002 | 0.0031 | 0.2131 |
| DanileGagnon | 28 | 0 | 0 | 0.0000 | 0.0000 | 0.0000 | 0.0000 | 0.0000 |
| dixonsociety | 29 | 4 | 7 | 0.0364 | 0.0636 | 0.0125 | 0.0006 | 0.3043 |
| DRCCca | 30 | 26 | 24 | 0.2364 | 0.2182 | 0.2092 | 0.0076 | 0.3719 |
| DWS_EndViolence | 31 | 4 | 3 | 0.0364 | 0.0273 | 0.0438 | 0.0000 | 0.3138 |
| emphasemcq | 32 | 2 | 1 | 0.0182 | 0.0091 | 0.0016 | 0.0001 | 0.2550 |
| EndingViolence | 33 | 30 | 1 | 0.2818 | 0.0091 | 0.1543 | 0.0000 | 0.4435 |
| EndViolenceBC | 34 | 19 | 20 | 0.1818 | 0.1909 | 0.0928 | 0.1052 | 0.4037 |
| endwomanabuse | 35 | 9 | 22 | 0.0818 | 0.2000 | 0.0905 | 0.0047 | 0.3252 |
| envisioncsc | 36 | 4 | 2 | 0.0364 | 0.0182 | 0.0123 | 0.0123 | 0.3188 |
| FemaideON | 37 | 6 | 7 | 0.0545 | 0.0636 | 0.0302 | 0.0293 | 0.3477 |
| FreeOfViolence | 38 | 27 | 10 | 0.2545 | 0.0909 | 0.1205 | 0.0567 | 0.4078 |
| fsjwrs | 39 | 0 | 0 | 0.0000 | 0.0000 | 0.0000 | 0.0000 | 0.0000 |
| ftplace | 40 | 4 | 5 | 0.0364 | 0.0455 | 0.0340 | 0.0007 | 0.3150 |
| gwwic | 41 | 26 | 23 | 0.2364 | 0.2182 | 0.2199 | 0.0304 | 0.3977 |
| Havre_Femmes | 42 | 1 | 0 | 0.0091 | 0.0000 | 0.0015 | 0.0000 | 0.2699 |
| HN_Women | 43 | 0 | 3 | 0.0000 | 0.0273 | 0.0000 | 0.0000 | 0.0000 |
| Hope24_7 | 44 | 3 | 5 | 0.0273 | 0.0455 | 0.0337 | 0.0000 | 0.3101 |
| HuroniaTHomes | 45 | 9 | 9 | 0.0818 | 0.0818 | 0.0810 | 0.0003 | 0.3150 |
| KausheesPlace | 46 | 1 | 0 | 0.0091 | 0.0000 | 0.0000 | 0.0000 | 0.0091 |
| Kawartha_SAC | 47 | 25 | 24 | 0.2273 | 0.2182 | 0.2060 | 0.0106 | 0.3881 |
| KenoraSAC | 48 | 7 | 11 | 0.0636 | 0.1000 | 0.0660 | 0.0000 | 0.3031 |
| KlinicCHC | 49 | 2 | 6 | 0.0182 | 0.0545 | 0.0107 | 0.0010 | 0.2789 |
| Ksacc_Ksacc | 50 | 3 | 3 | 0.0273 | 0.0273 | 0.0089 | 0.0000 | 0.2838 |
| KsanSociety | 51 | 2 | 3 | 0.0182 | 0.0273 | 0.0110 | 0.0000 | 0.3020 |
| Lachrysalide | 52 | 0 | 0 | 0.0000 | 0.0000 | 0.0000 | 0.0000 | 0.0000 |
| LaDauphinelle | 53 | 0 | 0 | 0.0000 | 0.0000 | 0.0000 | 0.0000 | 0.0000 |
| lelancalacs | 54 | 7 | 8 | 0.0636 | 0.0727 | 0.0001 | 0.0038 | 0.2131 |
| LSAIC | 55 | 4 | 4 | 0.0364 | 0.0364 | 0.0136 | 0.0000 | 0.2953 |
| MCS_Regina | 56 | 1 | 1 | 0.0091 | 0.0091 | 0.0003 | 0.0000 | 0.2142 |
| mpssas1 | 57 | 24 | 21 | 0.2182 | 0.1909 | 0.1952 | 0.0046 | 0.3554 |
| NEOSS_news | 58 | 0 | 1 | 0.0000 | 0.0091 | 0.0000 | 0.0000 | 0.0000 |
| NIAGARA_SAC | 59 | 30 | 24 | 0.2727 | 0.2182 | 0.2216 | 0.0118 | 0.3862 |
| NLSACPC | 60 | 7 | 5 | 0.0636 | 0.0455 | 0.0586 | 0.0003 | 0.3150 |
| NsCrisis | 61 | 0 | 0 | 0.0000 | 0.0000 | 0.0000 | 0.0000 | 0.0000 |
| OasisFemmes | 62 | 6 | 4 | 0.0545 | 0.0364 | 0.0342 | 0.0146 | 0.3375 |
| OCRCC_ON | 63 | 24 | 25 | 0.2182 | 0.2273 | 0.1953 | 0.0087 | 0.3539 |
| OptionsBC | 64 | 2 | 7 | 0.0182 | 0.0636 | 0.0022 | 0.0009 | 0.2487 |
| ORCCsupports | 65 | 25 | 33 | 0.2273 | 0.3000 | 0.2002 | 0.0377 | 0.4037 |
| PACECentreGP | 66 | 9 | 5 | 0.0818 | 0.0455 | 0.0477 | 0.0011 | 0.3292 |
| PCWomensCentre | 67 | 3 | 4 | 0.0273 | 0.0364 | 0.0047 | 0.0020 | 0.2542 |
| Pointdappui1 | 68 | 7 | 6 | 0.0636 | 0.0545 | 0.0001 | 0.0012 | 0.2131 |
| PrinceGeorgeSAC | 69 | 8 | 5 | 0.0727 | 0.0455 | 0.0679 | 0.0013 | 0.3492 |
| regina_rsac | 70 | 3 | 3 | 0.0273 | 0.0273 | 0.0054 | 0.0174 | 0.2848 |
| ReseaudesCAVAC | 71 | 4 | 3 | 0.0364 | 0.0273 | 0.0017 | 0.0123 | 0.2723 |
| RQCALACS | 72 | 10 | 14 | 0.0909 | 0.1273 | 0.0016 | 0.1422 | 0.2770 |
| SACCW_E | 73 | 6 | 8 | 0.0545 | 0.0727 | 0.0567 | 0.0013 | 0.3333 |
| sacetalks | 74 | 12 | 21 | 0.1091 | 0.1909 | 0.0758 | 0.0266 | 0.3570 |
| SACHA_tweets | 75 | 0 | 1 | 0.2182 | 0.2727 | 0.2044 | 0.0183 | 0.3685 |
| SACHAhamont | 76 | 24 | 30 | 0.0000 | 0.0091 | 0.0000 | 0.0000 | 0.0000 |
| sackingston | 77 | 28 | 34 | 0.2545 | 0.3091 | 0.2239 | 0.0327 | 0.4078 |
| SADVTC | 78 | 40 | 38 | 0.3636 | 0.3455 | 0.2467 | 0.1645 | 0.4725 |
| SaffronCentre | 79 | 6 | 6 | 0.0545 | 0.0545 | 0.0245 | 0.0005 | 0.3066 |
| SanareCentre | 80 | 7 | 7 | 0.0636 | 0.0636 | 0.0168 | 0.0009 | 0.2986 |
| SARAforWomen | 81 | 3 | 3 | 0.0273 | 0.0273 | 0.0016 | 0.0156 | 0.2518 |
| SascOttawa | 82 | 21 | 30 | 0.1909 | 0.2727 | 0.1803 | 0.0123 | 0.3477 |
| SASCSarnia | 83 | 14 | 10 | 0.1273 | 0.0909 | 0.1305 | 0.0004 | 0.3375 |
| SASCWR | 84 | 27 | 28 | 0.2455 | 0.2545 | 0.2132 | 0.0144 | 0.3754 |
| SAVISofHalton | 85 | 27 | 25 | 0.2455 | 0.2273 | 0.2188 | 0.0098 | 0.3789 |
| SCCommunityServ | 86 | 0 | 0 | 0.0000 | 0.0000 | 0.0000 | 0.0000 | 0.0000 |
| sesac_casse | 87 | 1 | 0 | 0.0091 | 0.0000 | 0.0001 | 0.0000 | 0.2131 |
| sosviolence | 88 | 1 | 4 | 0.0091 | 0.0364 | 0.0001 | 0.0008 | 0.2103 |
| StatusofWomenNT | 89 | 1 | 2 | 0.0091 | 0.0182 | 0.0133 | 0.0064 | 0.3113 |
| SurreyWomens | 90 | 8 | 12 | 0.0727 | 0.1091 | 0.0294 | 0.0290 | 0.3462 |
| SurvivorsHopeMB | 91 | 5 | 2 | 0.0455 | 0.0182 | 0.0353 | 0.0000 | 0.3448 |
| TAWCTimmins | 92 | 30 | 24 | 0.2727 | 0.2182 | 0.2229 | 0.0329 | 0.4414 |
| tearmannsociety | 93 | 4 | 3 | 0.0364 | 0.0273 | 0.0011 | 0.0030 | 0.2003 |
| THANS_NS | 94 | 5 | 8 | 0.0455 | 0.0727 | 0.0052 | 0.0205 | 0.2558 |
| THFCapeBreton | 95 | 0 | 0 | 0.0000 | 0.0000 | 0.0000 | 0.0000 | 0.0000 |
| ThirdPlaceTH | 96 | 6 | 4 | 0.0545 | 0.0364 | 0.0055 | 0.0049 | 0.2566 |
| trccmwar | 97 | 12 | 22 | 0.1091 | 0.2000 | 0.1216 | 0.0020 | 0.3279 |
| TriCityTS | 98 | 5 | 4 | 0.0455 | 0.0364 | 0.0133 | 0.0017 | 0.3054 |
| VanRapeRelief | 99 | 0 | 12 | 0.0000 | 0.1091 | 0.0000 | 0.0000 | 0.0000 |
| VSACentre | 100 | 13 | 11 | 0.1182 | 0.1000 | 0.0795 | 0.0094 | 0.3347 |
| Waypointswb | 101 | 1 | 3 | 0.0091 | 0.0273 | 0.0076 | 0.0000 | 0.2879 |
| WomenInCrisis1 | 102 | 6 | 13 | 0.0545 | 0.1182 | 0.0640 | 0.0001 | 0.3213 |
| WomensHouseBG | 103 | 7 | 12 | 0.0636 | 0.1091 | 0.0710 | 0.0003 | 0.3226 |
| WSACRenfrew | 104 | 14 | 16 | 0.1273 | 0.1455 | 0.1383 | 0.0082 | 0.3265 |
| WSNYorkRegion | 105 | 20 | 20 | 0.1909 | 0.1909 | 0.1846 | 0.0060 | 0.3477 |
| YWCABrandon | 106 | 1 | 1 | 0.0091 | 0.0091 | 0.0002 | 0.0000 | 0.2054 |
| YWCALethbridge | 107 | 4 | 7 | 0.0364 | 0.0636 | 0.0037 | 0.0194 | 0.2696 |
| YWCANWT | 108 | 2 | 4 | 0.0182 | 0.0364 | 0.0009 | 0.0051 | 0.2384 |
| YWCA_PH | 109 | 7 | 9 | 0.0636 | 0.0818 | 0.0566 | 0.0146 | 0.3418 |
| _hsls | 110 | 3 | 4 | 0.0273 | 0.0364 | 0.0255 | 0.0005 | 0.3126 |
